# Clinical Update Findings about pH-Impedance Monitoring Features in Laryngopharyngeal Reflux Patients

**DOI:** 10.3390/jcm11113158

**Published:** 2022-06-01

**Authors:** Jerome R. Lechien

**Affiliations:** 1 Polyclinic of Poitiers, Elsan Hospital, 86000 Poitiers, France; jerome.lechien@umons.ac.be; 2 Department of Anatomy and Experimental Oncology, Mons School of Medicine, UMONS Research Institute for Health Sciences and Technology, University of Mons (UMons), 7000 Mons, Belgium; 3 Department of Otolaryngology-Head and Neck Surgery, EpiCURA Hospital, 7301 Baudour, Belgium; 4 Department of Otolaryngology-Head and Neck Surgery, Foch Hospital, Paris Saclay University, 92150 Paris, France

**Keywords:** larynx, laryngitis, laryngopharyngeal, reflux, otolaryngology, head neck surgery, gastroesophageal reflux, pH impedance, monitoring, testing

## Abstract

Purpose: The diagnosis of laryngopharyngeal reflux (LPR) is commonly based on non-specific symptoms and findings and a positive response to an empirical therapeutic trial. The therapeutic response is, however, unpredictable, and many patients need pH-impedance monitoring to confirm the diagnosis. Methods: A review of the recent literature was conducted in *PubMED*, *Scopus*, and *Embase* about the pH-study features of LPR patients. A summary of last evidence was proposed. Results: The awareness of otolaryngologists about indications and interpretation of pH-impedance monitoring is low. The hypopharyngeal-esophageal multichannel intraluminal impedance-pH monitoring (HEMII-pH) is the most reliable examination determining the type and composition of hypopharyngeal reflux events (HRE) and the LPR features. The use of HEMII-pH is important to confirm the diagnosis in selected patients because non-specificity of symptoms and findings. There are no international consensus guidelines for the LPR diagnosis at the HEMII-pH. However, most studies supported the occurrence of >1 acid/weakly acid/nonacid HRE as diagnostic threshold. HREs are more frequently gaseous, weakly/nonacid compared with reflux events of gastroesophageal reflux. HREs occurred as daytime and upright, which does not support the value of double proton pump inhibitors or bedtime alginate. Oropharyngeal pH-monitoring is another approach reporting different sensitivity and specificity outcomes from HEMII-pH. The use of Ryan score for the LPR diagnosis at the oropharyngeal pH monitoring may be controversial regarding the low consideration of alkaline HREs. Conclusions: The awareness of otolaryngologists about HEMII-pH indication, features, and interpretation is an important issue regarding the high prevalence of LPR in outpatients consulting in otolaryngology. The HEMII-pH findings may indicate a more personalized treatment considering type and occurrence time of HREs.

## 1. Introduction

Laryngopharyngeal reflux (LPR) is an inflammatory condition of the upper aerodigestive tract tissues related to direct and indirect effect of gastroduodenal content reflux, which induces morphological changes in the upper aerodigestive tract [1]. The demonstration of pharyngeal reflux events through pH study is an important step in the management of LPR because this is the most effective approach to objective the back flow of gastric content into the pharynx [1]. The analysis of pH study features of LPR patients may improve the understanding of the LPR pathophysiological mechanisms [2]. The present review aims to summarize the current evidence about pH study findings of LPR patients.

## 2. Epidemiology

The prevalence of LPR remains unknown because lack of gold-standard procedure to assume the diagnostic [1,3]. Epidemiological studies reported prevalence of LPR-related symptoms in population ranging from 10% to 30% in Greece [4], the U.K. [5], China [6], and the USA [7], but symptoms considered for the suspicion of LPR in these studies were all non-specific and commonly found in many otolaryngological conditions (rhinosinusitis, infections, allergy, rhinitis, or tobacco-induced pharyngitis) [1,8,9,10,11]. The probable high prevalence of LPR and the non-specificity of symptoms make the use of objective examination important to strengthen the accuracy of diagnostic and treatment.

## 3. Diagnosis of Reflux and Place of pH Study

### 3.1. Clinical Diagnosis and Importance of pH Study

To date, most physicians consider patient symptoms and nasofibroscopic findings for the LPR check-up and confirm the diagnosis through symptom improvement after 1- to 3-month empirical therapeutic trial [12]. The use of patient-reported outcome questionnaires (e.g., reflux symptom index > 13 [13] or reflux symptom score > 13 [14]) and clinical instruments (e.g., reflux finding score > 7 [15] or reflux sign assessment > 14 [16]) improves the clinical diagnostic accuracy [1,17]. However, the clinical approach is limited for many reasons. First, the non-specificity of symptoms and findings makes uncertain the clinical diagnostic, and the prescription of empirical treatment may be inconsistent and costly [18]. Second, the empirical therapeutic success remains uncertain, with 57% of patients reporting improvement or relief of symptoms [18]. The use of inadequate treatment, the chronic course of some LPR disease presentations, and the patient adherence are all factors that may underly the low empirical therapeutic success rate [18]. According to recent reviews [18,19], most authors used proton pump inhibitors (PPIs) for the empirical treatment, even though most hypopharyngeal reflux events (HREs) are weakly or nonacid at the hypopharyngeal-esophageal multichannel intraluminal impedance–pH monitoring (HEMII-pH) [20,21,22]. The use of PPIs with alginate or magaldrate makes further sense [18], but this combination remains infrequently used [23,24]. The low success rate of empirical treatment may result from the clinical course of some LPR diseases. Indeed, LPR may be acute (30%), recurrent (40%), or chronic (30%) disease [25]. Patients with chronic course reported low therapeutic response rates for unknown reasons [25,26]. Another issue that may underly the difficulty to reach adequate therapeutic success rate is the lack of adherence of patients to treatment regimen [27]. In practice, many patients did not experience heartburn or gastroesophageal reflux disease (GERD)-related symptoms and may doubt the reflux (LPR) diagnosis, which may strengthen the poor therapeutic adherence. The patient fear about suspected adverse effects of PPIs is another barrier for patient adherence [27]. The diagnosis of LPR may be improved with pH-impedance monitoring in significant cases. De Bortoli et al. observed that the LPR diagnosis was not confirmed at the pH-impedance monitoring in about 40% of cases that were clinically diagnosed with reflux laryngitis [28].

In sum, the clinical diagnosis based on empirical therapeutic trial is currently considered as a reasonable first-line strategy, but many patients may not report symptom relief and may require objective examination to confirm the LPR diagnosis.

### 3.2. Place, Indications, and Features of pH Study

To date, there is no objective tool considered as the gold standard for the LPR diagnostic. According to the characteristics of the device (impedance ring, placement of sensors, etc.), the pH study may be considered as the most reliable tool to demonstrate the back flow of gastric content into the pharynx. This approach is associated with advantages and disadvantages that may be considered in the decision of physician to propose pH study to the patients.

The pH study is usually performed over a 24-h period, which may be associated with patient inconvenience despite adequate tolerance [29]. Most patients tolerate the examination (>95%) [29]. The catheter insertion may be associated with significant pain, and the pH probe may cause belching and coughing during the early part of the monitored period, especially in patients with esophageal or laryngopharyngeal mucosa hypersensitivity [29,30]. The pharyngeal probe placement difficulties and movements are both points that were frequently considered as weaknesses of the technique, leading to probe movement and false-positive diagnostic [29,31]. From a theoretical standpoint, it has for a long time been suggested that drying of the hypopharyngeal sensors led to pseudoreflux and false positive, but in practice, this was not really demonstrated [31].

The main advantage of pH study is the identification of HREs and their following features: composition (gaseous, liquid versus mixed), types (acid, weakly acid, versus nonacid), and the position of occurrence (upright versus supine). The identification of the LPR features may lead to a more personalized treatment considering the usefulness of PPIs (acid/weakly acid versus alkaline reflux) as well as the time of medication intake (daytime, nighttime or 24-h reflux) [32]. In other words, pH study may be useful for the therapeutic strategy.

To date, there are no consensus for the indications of HEMII-pH. According to a recent survey, most otolaryngologists do not prescribe pH study and prefer to refer patients to the gastroenterologist for the following reasons: patient inconvenience (59.4%), lack of understanding of interpretation (49.2%), lack of meaningfulness (42.8%), lack of skills to interpret the results (35.4%), and the suspected high cost of the approach (35.1%). Among aware otolaryngologists, HEMII-pH was mainly proposed to resistant patients for an empirical therapeutic trial [23,24].

### 3.3. Single, Dual-, or Triple-Probe Esophageal pH Monitoring

The consideration of LPR as a different condition than GERD appeared in the nineties with the work of Jamie Koufman [26,33]. In 1991, Jamie Koufman estimated the LPR incidence at 10% of outpatients presenting to otolaryngology departments with extra-esophageal manifestations of GERD [26]. In this study, 62% of individuals had abnormal esophageal pH studies considering acid GERD criteria, and 30% reported documented acid reflux events in both esophagus and pharynx. This study was perhaps the first important research differentiating LPR from GERD, but the dual-probe pH study device only focused on acid HRE.

Triple-probe hypopharyngeal-esophageal pH monitoring was used in many studies over the two last decades considering the LPR diagnostic when pharyngeal drop in pH value <4 occurred immediately after distal and proximal esophageal acid exposure [31,34,35]. The use of triple-probe pH study provided new information in LPR physiology about the role of upper esophageal sphincter (UES). Initially, Murris et al. observed that 24% of LPR patients may have acid HRE but normal acid exposure in the low esophagus [31]. These authors also reported that only 68% of proximal esophageal reflux events reached pharynx [31]. The lack of association between distal esophageal and pharyngeal acid events was corroborated by Postma et al. who observed that 38% of LPR patients (>1 pharyngeal acid event) had normal esophageal acid exposure times [36]. Interestingly, Harrel et al. observed that adding a hypopharyngeal pH sensor in pH study increased the detection of abnormal pH values and supported the diagnosis of LPR more often than traditional dual-sensor esophageal monitoring [34]. Nowadays, the accuracy of single, dual-, or triple-probe pH-study devices is called into question regarding the lack of correlation between distal/proximal esophageal events and HREs and the lack of consideration of weakly acid or nonacid HREs [20,21,22,37].

### 3.4. Multichannel Intraluminal Impedance–pH Monitoring

The recent literature dedicated to MII-pH without pharyngeal sensor was not reviewed because only HEMII-pH may detect HRE. To date, there is no international consensus guidelines determining HRE threshold for the LPR diagnosis. In a recent study, Kim et al. observed that the consideration of ≥1 HRE at the HEMIII-pH was associated with sensitivity and specificity of 76.0% and 81.5%, respectively [38]. According to the type of pH-impedance monitoring used for the diagnosis, the diagnosis criteria may vary. Many differences across studies make difficult the establishment of consensual normative criteria for LPR on ambulatory reflux monitoring, e.g., impedance/pH sensor placements or configurations, definition of HRE, definition of composition (gas, liquid, mixed), or type (acid/weakly acid/nonacid) events (Table 1 and Table 2) [37,39,40,41,42,43,44,45,46,47,48,49,50,51,52,53]. These differences may involve the various devices available on the market. Thus, in a recent systematic review including the pH study findings of 720 healthy individuals, authors observed that the 95th percentile thresholds were 0 to 10 HREs for HEMII-pH and 40 to 128 for events with pH < 6.0 on oropharyngeal pH monitoring, respectively [37]. These differences between HEMII-pH and oropharyngeal pH monitoring may be related to different sensitivities and precisions of pH study devices in the HRE detection.

**Table 1:** Definitions of hypopharyngeal reflux events according to studies.

Hypopharyngeal Reflux Event Definition and Features	References
1. Episode reaching proximally to 1 cm above the upper border of UES (with decreased impedance).	[39,40]
2. Episode reaching proximally to 0.5 cm above the upper border of UES (with decreased impedance).	[41]
3. Retrograde 50% drop in impedance starting distally (UES) and reaching the more proximal impedance site. HRE event was considered only if it was preceded by retrograde impedance drop both distally and proximally within the esophagus and if no swallow occurred during the pharyngeal impedance drop.	[42]
4. Episode when the time of pH reaching to the lowest point was no more than 30 s (Restech).	[43]
5. Reflux reaching Z1 Z2 (hypopharyngeal) impedance segment.	[44,45]
6. Episode reaching oropharyngeal sensor.	[46,47,48,49]

In study assessing the normative data for reflux patients, six definitions of hypopharyngeal reflux event were used. Abbreviations: HRE, hypopharyngeal reflux event; UES, upper esophageal sphincter.

**Table 2:** Type and composition definitions of reflux event according to studies.

Outcomes	Definition and Features	References
**Reflux Event Composition**		
Gas HRE	1. Simultaneous increase in impedance of >3000 W in any two consecutive impedance sites with one site with an absolute value >7000 W in the absence of swallowing.	[39,40,41,42,44,45,50,51,52]
	2. Abrupt increase of impedance by ≥50% in two adjacent channels with simultaneous or near-simultaneous propagation in the retrograde direction.	[53]
Liquid HRE	1. Retrograde 50% drop in impedance starting distally (LES) and propagating at least to the next two or more proximal impedance measuring segments.	[39,40,41,42,44,45,50,51,53]
	2. Retrograde moving 40% fall in impedance in two distal impedance sites.	[52]
Mixed HRE	1. Gas reflux occurring immediately before or during a liquid reflux.	[39,40,41,42,45,50]
	2. Combination of the gas reflux and liquid reflux patterns.	[44,53]
	3. <50% fall in impedance of resting impedance (liquid) preceded or followed by an abrupt rise in impedance (gas).	[51]
**Reflux Event Types**		
Acid HRE/PRE	1. Hypopharyngeal or proximal esophageal event with pH < 4.	[39,40,44,50]
	2. Hypopharyngeal and proximal esophageal events with pH < 4.	[41]
	3. Drop/event in pH < 4 for at least 5 s in the proximal esophagus.	[52,53]
	4. Drop/event in pH < 4.0 from a pre-event pH > 4.0 units lasting for >5 s.	[40,51]
Superimposed acid PRE	1. Reflux event while pH < 4 during an acid clearing interval.	[50]
	2. Liquid reflux monitored by impedance electrodes while esophageal pH is still <4.0.	[40,51]
Weakly acid HRE/PRE	1. Hypopharyngeal or proximal esophageal pH 4–7.	[39,40,50,51]
	2. Hypopharyngeal and proximal esophageal pH > 4.	[41]
	3. Decrease of more than 1 pH unit with a nadir pH above 4.	[53]
Nonacid HRE/PRE (Weakly alkaline)	1. Hypopharyngeal or proximal esophageal pH > 7.	[39,40,41,50]
	2. No change of pH or a decrease of less than 1 pH unit.	[53]
	3. Hypopharyngeal or proximal esophageal pH > 4.	[44]
	4. Hypopharyngeal or proximal esophageal pH ≥ 7.0.	[40,51]

Abbreviations: LES, lower esophageal sphincter; HRE = hypopharyngeal reflux event.

**Figure 1:**
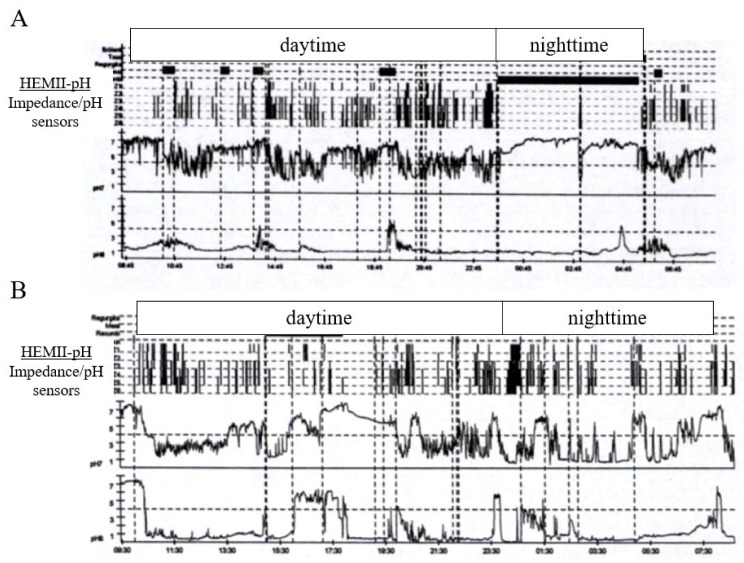
pH-impedance profiles of patients with gastroesophageal reflux disease and laryngopharyngeal reflux. Two main profiles of LPR patients at the HEMII-pH are observed: patients with daytime hypopharyngeal reflux episodes (**A** most of cases) and patients with daytime and nighttime hypopharyngeal reflux episodes (**B**). Abbreviations: GERD, gastroesophageal reflux disease; HEMII-pH, hypopharyngeal-esophageal multichannel intraluminal impedance–pH monitoring; LPR, laryngopharyngeal reflux.

The profiles of LPR patients at the HEMII-pH were studied in few studies. It was found in a recent study that 74% of HREs occurred outside 1-h post-meal times, while 20.5% and 5.5% occurred during the 1-h post meal and nighttime, respectively (Figure 1) [22]. LPR was nonacid or weakly acid in more than half patients, and they had only upright and daytime HREs in 59% of cases [22]. The findings of this study corroborated those summarized in the systematic review of the Young Otolaryngologists of the International Federation of Otorhinolaryngological Societies [37]. The occurrence of daytime, upright, and gaseous HREs involved esophageal dysmotility, especially transient relaxations of the lower and upper esophageal sphincters. Thus, Sikavi et al. observed that LPR patients (with or without coexisting motility disorder) had reduced proximal esophageal contractibility at the high-resolution manometry, which significantly predicted increased of HREs [2]. The same team reported in another publication that 43.3% of patients with LPR at the HEMII-pH had abnormal findings at the high-resolution manometry, with the ineffective esophageal motility being the most common diagnosis [54]. Interestingly, recent findings reported that most HREs are weakly or nonacid [6,38,41,55], which supports the consideration of alginate or magaldrate in the therapeutic strategy [32,55]. Moreover, in practice, the pH of the reflux event may increase from the distal to the proximal esophagus. The mechanisms underlying this increase of pH remain unknown and would involve the bicarbonate secretion into the esophagus mucosa. The HEMII-pH features of LPR versus GERD patients are summarized in Table 3.

### 3.5. Oropharyngeal pH Monitoring

Oropharyngeal pH monitoring (Restech Dx–pH monitoring) was specifically developed for the diagnosis of LPR [48]. As for HEMII-pH studies, there are several diagnostic criteria in the literature [37]m but many authors agreed to consider a positive Ryan score (upright score ≥ 9.41 or supine score ≥ 6.8) for the presence of LPR [46,48,49,56,57]. Ryan score is calculated according to three components: the percent time pH < 5.5 upright or <5.0 supine; the number of episodes in which the pH dropped below threshold; and the duration of the longest episode. The low consideration of HRE with pH > 7.0 in the Ryan score is a controversial issue because many studies demonstrated that there are significant proportions of HREs with pH > 7 in LPR patients [22,37,38]. At alkaline pH, the bile salts and some potential other enzymes may injury the laryngopharyngeal mucosa. Interestingly, Vance et al. compared the diagnostic utility of HEMII-pH study versus oropharyngeal pH monitoring in patients who benefited from both examinations throughout the same 24-h period [58]. These authors reported that oropharyngeal pH monitoring (Restech^®^) detected more percent time/total HREs in supine and upright positions and longer event times compared with HEMII-pH. Moreover, HEMII-pH testing was able to detect more HREs of pH < 4 than oropharyngeal pH monitoring [58]. Vance et al. observed that oropharyngeal pH monitoring correlated better with total patient symptom scores including cough, heartburn, burping, and throat clearing compared with HEMII-pH. The findings of Vance et al. do not corroborate those of Weitzendorfer et al. who observed that elevated Dx-pH measurements did not show significant correlation with either pH–impedance monitoring features, RSI, RFS, and saliva pepsin measurements [59]. However, irrespective to the pH study device, the correlation between pH study, symptoms, and findings remains controversial according to many studies that could not demonstrate an apparent relationship between the intensity of symptoms and the magnitude and patterns of hypopharyngeal reflux events [1,60]. This lack of correlation may be related to various patient profiles of mucosa sensitivity and microbiome differences [61]. In sum, the usefulness of oropharyngeal pH monitoring needs to be demonstrated in future controlled studies.

### 3.6. Placement and Technical Point

The placement of the pH study probe is commonly performed by an experienced nurse or physician who needs to be aware about potential complications, including probe kink or pulmonary placement [62]. In HEMII-pH, the distal sensor is usually placed 5 cm above the upper margin of the lower esophageal sphincter to avoid displacement into the stomach during swallowing, when the esophagus is shortened [31]. The pharyngeal sensor is placed 1–2 cm above the UES, but this position may change from one study to another [37]. In oropharyngeal pH monitoring, the pharyngeal sensor is placed in the oropharynx cavity. The control of the HEMII-pH probe placement may be done with chest radiography, nasofibroscopy, or pH control in the distal sensor (stomach). Importantly, the analysis of HEMII-pH needs to be performed by experienced otolaryngologist or gastroenterologist because automated analysis was found to be associated with a tendency of excessive reflux measurement when compared with manual analysis [63].

## 4. Perspective of pH Study in Otolaryngology

According to the potential associations between LPR and many otolaryngological disorders, including suppurative otitis media [64,65], recalcitrant chronic rhinosinusitis [66], some benign lesions of the vocal folds [56,67,68], nonfunctional laryngeal disorders [69], vocal fold granuloma [70], Eustachian tube dysfunction [71], eye dryness [72], or chronic nasal obstruction [73,74], the usefulness of pH study in otolaryngology is an important issue. The identification of LPR as contributing factor of the above-mentioned otolaryngological conditions may lead to better therapeutic regimen and control of the clinical courses of the diseases. In sinonasal, Eustachian tube, or otological disorders, the pH study may detect reflux events in nasopharyngeal cavity, which involves nasopharyngeal pH sensors [75]. In that way, some nasopharyngeal pH monitoring devices were developed [76], but they did not detect nonacid or weakly acid reflux events, which was their primary limitation. The usefulness of oropharyngeal pH monitoring for these disorders would make particularly sense [75]. Indeed, the sensor of oropharyngeal pH monitoring may be placed in nasopharynx to detect acid, whether weakly acid and nonacid reflux events [75]. The study of LPR in the development of common otological and sinonasal diseases is a future research topic in otolaryngology. However, the awareness of otolaryngologists about LPR and pH study needs to be improved. Currently, only 5% of otolaryngologists were aware about the indication and usefulness of pH study [23,24]. In the U.S. and Eastern Asia, 10% of otolaryngologists recognized using (HE)MII-pH, while they were 6.6%, 6.2%, and 2.0% in Europe, South America, and West Asia/Africa, respectively [23]. The relationships between LPR and sinonasal or otological conditions were known by less than 50% of responders [23]. Similar findings were observed in other surveys, including among European [77], Asian [78], or South American [79] otolaryngologist populations. The use of pH–impedance monitoring in these new indications as well as the consideration of new pH–impedance metrics (i.e., the mean nocturnal baseline impedance and post-reflux swallow-induced peristaltic wave index) are future issues to explore in the next few years [80].

Pepsin saliva detection (Peptest^®^; RD Biomed Ltd., Hull, UK) is another diagnostic method that was investigated in the past decade [81,82]. Pepsin is involved in the development of mucosal inflammatory reaction, and injuries and may be detected in saliva of LPR patients [1]. According to a recent systematic review, the pooled sensitivity and specificity for the diagnosis of LPR with Peptest^®^ (>16 ng/mL) were 62% and 74%, respectively [83]. Zhang et al. assessed the sensitivity, specificity, and predictive values of Peptest^®^ regarding different thresholds of saliva pepsin measurements [84]. These authors observed that the use of 75 ng/mL in place of 16 ng/mL for the diagnosis of LPR decreased the sensitivity from 76.9% to 57.7%, while the positive predictive value increased from 87% to 93.8% [84]. In sum, the pepsin saliva measurement is another diagnostic approach with less reliability than HEMII-pH.

## 5. Conclusions

The HEMII-pH may help the otolaryngologist to confirm and treat the LPR disease. pH studies may be indicated for resistant patients to empirical therapeutic trial or those with a chronic course of the disease. The awareness of otolaryngologists about HEMII-pH indications, features, and interpretation is an important issue because they may indicate a more personalized treatment considering type and occurrence time of HRE. Future studies are needed to explore the usefulness of oropharyngeal pH monitoring and the potential indications in some otolaryngological conditions associated with reflux.

## Figures and Tables

**Table 3:** pH–impedance differences between gastroesophageal reflux disease and laryngopharyngeal reflux.

Impedance–pH Monitoring Features		
Outcomes	GERD	LPR
Distal esophageal events	Large number of acid episodes	May be normal
	High acid exposure	
	Diagnostic criteria *	
Proximal/pharyngeal events	Infrequent	>1 events
	Acid events (if present)	Weakly/nonacid events
Composition of reflux	Mainly liquid	Mainly gaseous
Time of events	Supine & upright	Upright
Favoring factor	Supine position	-
Types of reflux	Mainly acid	Mainly weakly/nonacid
Correlation between	Frequently significant	Rarely significant
Symptoms–events		

* GERD diagnosis is based on acid exposure in the low esophagus. GERD/LPR patients reported higher proportions of acid proximal/pharyngeal events than LPR patients only. Both refluxes are characterized by different positional effects. The correlation between symptom–reflux events is often non-significant in LPR patients due to the tardiness of refluxate effects on the clinical pattern of disease. Abbreviations: GERD, gastroesophageal reflux disease; LPR, laryngopharyngeal reflux.

## Data Availability

Not applicable.
